# Factors associated with polyacrylamide hydrogel outcomes in women with stress urinary incontinence

**DOI:** 10.1002/bco2.218

**Published:** 2023-01-25

**Authors:** Venetia Hoe, Henry H. Yao, Karla Gough, Helen E. O'Connell

**Affiliations:** ^1^ Department of Urology Western Health Footscray Victoria Australia; ^2^ Department of Surgery The University of Melbourne Melbourne Victoria Australia; ^3^ Department of Health Services Research Peter MacCallum Cancer Centre Melbourne Victoria Australia; ^4^ Department of Nursing The University of Melbourne Melbourne Victoria Australia

**Keywords:** bladder compliance, Bulkamid®, outcomes, polyacrylamide hydrogel, stress urinary incontinence, urethral hypermobility

## Abstract

Knowledge of factors associated with superior outcomes in women treated with urethral bulking agents for stress urinary incontinence (SUI) remains limited. The aim of this study was to examine associations between post‐treatment outcomes in women who had undergone polyacrylamide hydrogel injections for SUI, and physiological and self‐reported variables captured during pre‐treatment clinical evaluation. A cross‐sectional study was undertaken in female patients treated for SUI with polyacrylamide hydrogel injections by a single urologist between January 2012 and December 2019. Post‐treatment outcome data were gathered in July 2020 using the Patient Global Impression of Improvement (PGI‐I), Urinary Distress Inventory‐short form (UDI‐6), Incontinence Impact Questionnaire (IIQ7), and International Consultation on Incontinence Questionnaire Short Form (ICIQ SF). All other data were gathered from women's medical records including pre‐treatment patient‐reported outcomes. Associations between post‐treatment outcomes and pre‐treatment physiological and self‐reported variables were investigated using regression models. One hundred seven of the 123 eligible patients completed post‐treatment patient‐reported outcome measures. Mean age was 63.1 years (range 25–93 years), and median time between first injection and follow‐up was 51 months (inter‐quartile range 23.5–70 months). Fifty‐five (51%) women had a successful outcome based on PGI‐I scores. Women with type 3 urethral hypermobility pre‐treatment were more likely to report treatment success (PGI‐I). Poor bladder compliance pre‐treatment was associated with greater urinary distress, frequency and severity (UDI‐6 and ICIQ) post‐treatment. Older age was associated with worse urinary frequency and severity (ICIQ) post‐treatment. Associations between patient‐reported outcomes and time between first injection and follow‐up were trivial and not statistically significant. Severity of pre‐treatment incontinence impact (IIQ‐7) was associated with worse incontinence impact post‐treatment. Type 3 urethral hypermobility was associated with a successful outcome, whereas pre‐treatment incontinence impact, poor bladder compliance and older age were associated with poorer self‐reported outcomes. Long‐term efficacy appears to hold in those who responded to initial treatment.

## 1 INTRODUCTION

The use of urethral bulking agents is a well‐established treatment in women with stress urinary incontinence (SUI).^1^ Normal urethral function includes coaptation of the mucosal folds to maintain a closed urethra at rest and during filling.^2^, ^3^ Injecting a bulking agent into the submucosal tissues of the urethra helps to restore loss of this coaptation function. Although generally regarded a second line surgical treatment option due to its lower efficacy compared with tension‐free vaginal tape and pubovaginal sling surgery, the increasing concerns of mesh morbidity associated with sling surgery have resulted in more women choosing to undergo urethral bulking treatment as a minimally invasive alternative for SUI.

Success rates of polyacrylamide hydrogel (Bulkamid®, Contura International A/S, Denmark) urethral bulking injection therapy are highly variable, ranging from 30% to 90% at 1 to 2 years of follow‐up.^4^ Evidence suggests this may be related to heterogeneity of patient cohorts, injection techniques and the definitions of treatment success used across various studies.^4^ Moreover, there are limited data on those most likely to benefit from urethral bulking.^1^, ^5^, ^6^ Knowledge of factors associated with superior outcomes could help clinicians better select and counsel patients on expected outcomes. The aim of this study was to examine associations between post‐treatment outcomes in women who had undergone polyacrylamide hydrogel injections for SUI, and physiological and self‐reported variables captured during pre‐treatment clinical evaluation.

## 2 METHODS

### 2.1 Design, setting and participants

We undertook a cross‐sectional study with retrospective case note review in female patients who underwent transurethral polyacrylamide hydrogel injections for the treatment of SUI by a single urologist at a large metropolitan hospital in Australia between January 2012 and December 2019.^7^ Ethical approval was obtained from the institutional HREC (EH2020‐621).

### 2.2 Variables and data sources

Each patient was clinically evaluated with a complete history and physical examination at the initial consultation. All patients had exertion‐related leakage and demonstrable stress incontinence with or without a urodynamic catheter in situ. Patient‐reported outcomes were also gathered at this time. Clinical and fluoroscopic urodynamic assessments were performed to confirm and sub‐type the diagnosis of SUI, as well as to rule out significant concomitant pelvic organ prolapse or other conditions requiring surgical treatment. Urethral mobility was classified as normal, type 1, type 2 or type 3 stress incontinence according to the degree of change of the proximal urethra and bladder neck during straining manoeuvres observed during fluoroscopy study.^2^

Pre‐treatment clinical and demographic data were extracted from medical records including age, comorbidities, previous anti‐incontinence procedures, number of injections received and pre‐treatment urodynamic parameters such as bladder neck (closed, open), abdominal leak point pressure (ALPP)^2^ and cough stress testing with and without 7F dual sensor TDOC® Urodynamic catheter at 250 cc, urethral hypermobility (non‐type 3 and type 3), maximal closing pressure (MUCP), and bladder compliance (normal and poor). Pre‐treatment patient‐reported outcomes data included responses to the Incontinence Impact Questionnaire Short Form (IIQ‐7)^8^ and Urinary Distress Inventory Short Form (UDI‐6)^8^ questionnaires (details provided below). ALPP testing was performed at 45‐degree upright position at 250 cc of filling in the absence of a detrusor pressure rise. Patients in whom no leak was seen during ALPP testing but in whom leakage was seen on direct visual examination with retraction of the posterior vaginal wall after catheter removal were sub‐categorised as ‘stress leak without catheter (SLWC)’. Some patients with a wide‐open bladder neck and severe hypermobility opted to undergo sling surgery from outset. The number of this category of SUI patient was not studied.

A cross‐sectional study was conducted by the clinic in July 2020. All patients who underwent polyacrylamide hydrogel injection at the private practice were contacted for follow‐up with post‐treatment outcome measures. Eligible patients were administered the UDI‐6, IIQ‐7, International Consultation on Incontinence Questionnaire Short Form (ICIQ‐SF)^9^ and Patient Global Impression of Improvement (PGI‐I).

The 6‐item UDI‐6 provides an overall measure of symptom distress associated with urinary incontinence symptoms. Respondents use a 4‐point Likert‐type scale ranging from ‘0’ (*not at all*) to ‘3’ (*greatly*). A total score is calculated for each respondent by averaging responses to items, then multiplying the average by 25. The possible range for the total score is 0 to 100, with higher scores reflecting higher levels of urinary distress.

The 7‐item IIQ‐7 provides a measure of the impact of urinary incontinence. Respondents use a 4‐point Likert‐type scale ranging from ‘0’ (*not at all*) to ‘3’ (*greatly*). A total score is calculated for each respondent by averaging responses to items, then multiplying the average by 33.3. The possible range for the total score is 0 to 100, with higher scores reflecting higher levels of incontinence impact.

The 4‐item ICIQ‐SF provides a measure of the frequency, severity and impact on quality of life associated with urinary incontinence. Two items use a Likert‐type scale, one ranging from ‘0’ (*never*) to ‘5’ (*all the time*), the other ranging from ‘0’ (*none*) to ‘6’ (*a large amount*). The final item uses an 11‐point numeric rating scale ranging from ‘0’ (*not at all*) to ‘10’ (*a great deal*). Responses are summed to create a total score with a possible range of 0 to 21, with higher scores reflecting higher levels of urinary severity, frequency and impact on quality of life.

The PGI‐I provides a measure of perceived improvement. Respondents use a 7‐point Likert scale ranging from ‘1’ (*very much better*) to ‘7’ (*very much worse*).^10^ The PGI‐I has been previously used to determine the success of incontinence procedures.^10^ Consistent with the definition employed in the Value of Urodynamic Evaluation (VALUE) study,^11^ treatment success was defined as a PGI‐I score of ≤ 2 (i.e., *very much better* or *much better*).

### 2.3 Statistical analysis

Descriptive statistics were used to summarise sample characteristics including treatment success based on PGI‐I scores. Logistic regression was used to model the probability of treatment success (reference group: failure). This was done separately for each pre‐treatment physiological and patient‐reported outcome variable. Linear regression was used to examine the relationship between pre‐treatment variables, and post‐treatment UDI‐6, IIQ‐7, and ICIQ‐SF scores; again, this was done separately for each pre‐treatment variable. Pre‐treatment variables included age (in years), UDI‐6 (in treatment success and UDI‐6 models), IIQ‐7 scores (in treatment success and IIQ‐7 models), bladder neck (closed and open), abdominal leak point pressure (ALPP: leak and SLWC), urethral hypermobility (non‐type 3 and type 3), mid‐urethral closing pressure (MUCP), and bladder compliance (normal and poor). UDI‐6 and IIQ‐7 scores were included in treatment success models. Early analysis showed no correlation between ALPP measurement at outcome. To permit regression analysis, a decision was made to convert ALPP categories into leak (demonstrated with catheter in situ) and no leak. Models including adjustment for time between first injection and follow‐up were also investigated. All analyses were performed using R (version 3.6.1) (R Core Team [2019]), with *p* < 0.05 (two‐sided) considered statistically significant.

## 3 RESULTS

**FIGURE 1:**
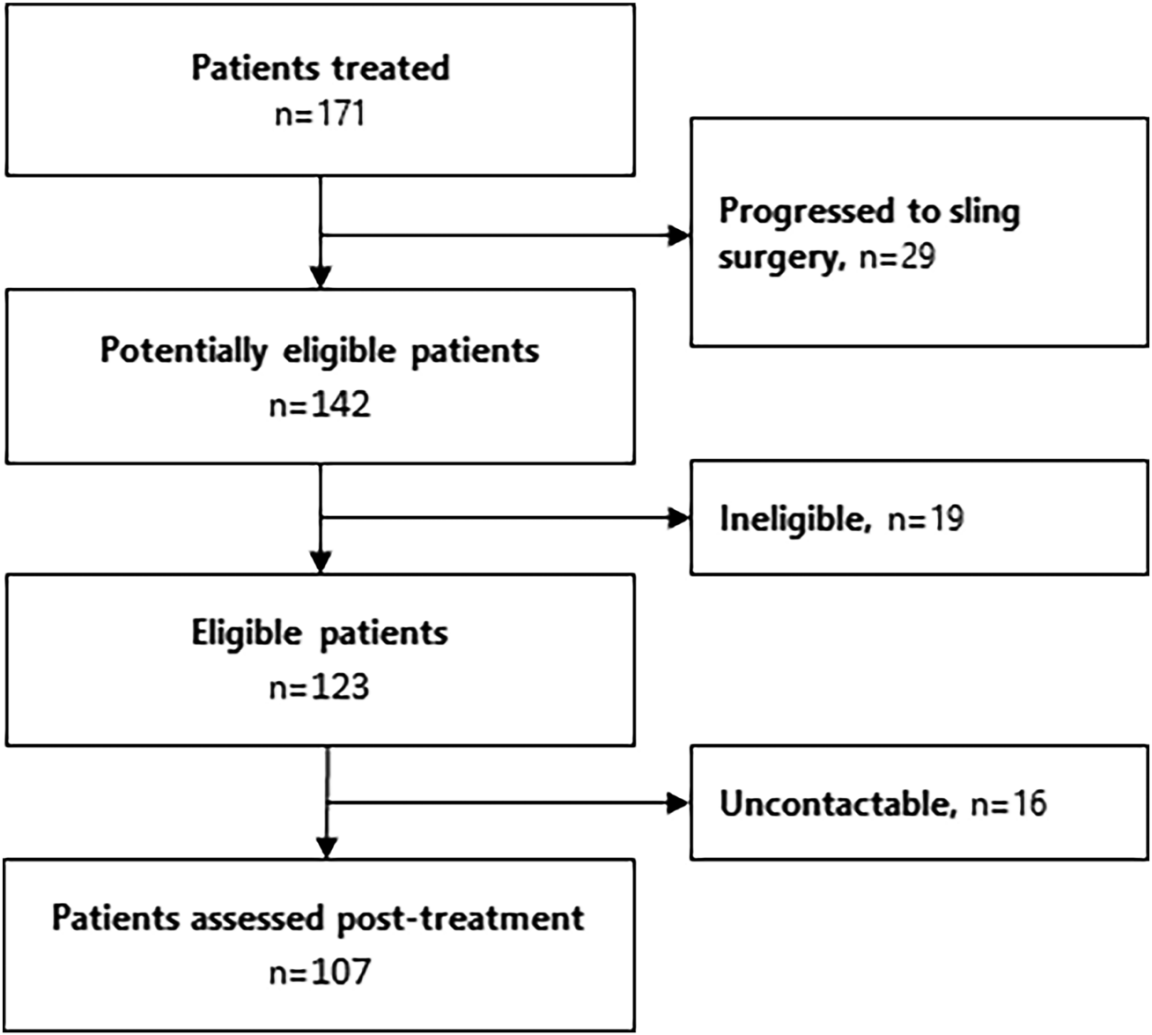
Study flow chart

**FIGURE 2:**
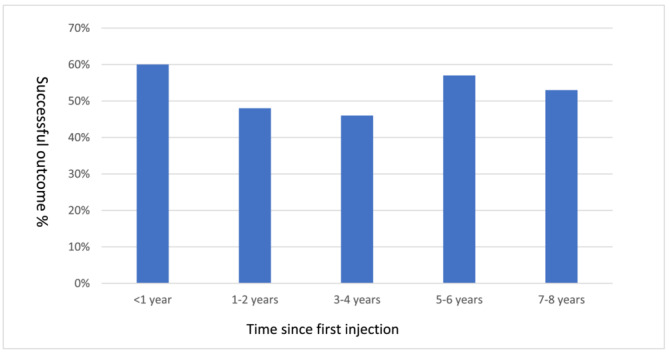
Descriptive statistics for successful outcome and time since first polyacrylamide hydrogel injection

One hundred twenty‐three of 171 women were eligible to participate in post‐treatment assessments (see Figure 1). Of these 123 women, 107 were contactable and consented to participate. Characteristics of the study sample are summarised in Table 1. Mean age was 63.1 years (range 25 to 93 years) and median time between first injection and follow‐up was 51 months (interquartile range 23.5 to 70). Using the pre‐specified cut‐off for success on the PGI‐I, 55 (51%) women had a successful outcome. Success rates by time since initial injection were of 6 of 10 (60%) at <1 year, 14 of 29 (48%) at 1–2 years, 12 of 26 (46%) at 3–4 years, 13 of 23 (57%) at 5–6 years, and 10 of 19 (53%) at 7–8 years (see Figure 2).

**TABLE 1:** Sample characteristics including pre‐ and post‐treatment patient‐reported outcomes (*n* = 107)

Variable	
Age (years)	
Mean	63.1
Standard deviation	13.3
Range	25 to 93
Follow‐up duration (months)	
Median	51
Interquartile range	23.5 to 70
Previous anti‐incontinence treatment, *n* (%)	39 (36%)
Urethral bulking agent (other than PAHG)	16 (15%)
Sling surgery	24 (22%)
Suspension surgery	7 (7%)
Other	3 (3%)
Number of PAHG injections, *n* (%)	
1	43 (40%)
2	64 (60%)
Treatment success, *n* (%)	55 (51%)
<1 year	6 (60%)
1–2 years	14 (48%)
3–4 years	12 (46%)
5–6 years	13 (57%)
7–8 years	10 (53%)

Abbreviation: PAHG, polyacrylamide hydrogel.

Logistic regression results for treatment success based on PGI‐I scores are summarised in Table 2, and linear regression results for post‐treatment UDI‐6, IIQ‐7, and ICIQ‐SF scores are summarised in Table 3. Results from the models adjusted for time between first injection and follow‐up were compatible with results from the unadjusted models; associations between post‐treatment patient‐reported outcomes and time between first injection and follow‐up were trivial and not statistically significant (results are available from the authors).

**TABLE 2:** Logistic regression results for PGI‐I scores (or treatment success)

Pre‐treatment variable	PGII^a^		OR (95% CI)^b^ ^,^ ^c^	*p*‐value
	Failure (*n* = 52)	Success (*n* = 55)		
Age	61.3 (14.4)	64.9 (12.2)	1.02 (0.99 to 1.05)	0.16
UDI‐6	45.9 (17.5)	43.4 (18.7)	0.99 (0.97 to 1.02)	0.52
IIQ‐7	30.7 (21.4)	29.6 (21.0)	0.99 (0.98 to 1.02)	0.80
Bladder neck				
Open	33 (47%)	37 (53%)	1.49 (0.62 to 3.68)	0.37
Closed	16 (57%)	12 (43%)		
ALPP				
Leak	34 (53%)	30 (47%)	0.79 (0.35 to 1.79)	0.57
Stress leak without catheter	17 (47%)	19 (53%)		
Urethral hypermobility				
Type 3	3 (20%)	12 (80%)	4.5 (1.32 to 20.79)	0.027
Non‐type 3	45 (53%)	40 (47%)		
MUCP	72.7 (33.6)	77.5 (39.6)	1.00 (0.99 to 1.02)	0.51
Bladder compliance				
Poor	21 (55%)	17 (45%)	0.71 (0.31 to 1.60)	0.41
Normal	29 (47%)	33 (53%)		

^a^ Data are means and standard deviations for age, UDI‐6 and IIQ‐7, and MUCP. Data are counts and percentages for bladder neck, ALPP, urethral hypermobility and bladder compliance.

^b^ Reference group: failure.

^c^ Odds ratios interpreted as follows: 0.82 (or 1.22), small‐sized association; 0.54 (or 1.86), medium‐sized association; and 0.33 (or 3.00), large‐sized association.^12^

**TABLE 3:** Linear regression results for post‐treatment patient‐reported outcomes

Measure and pre‐treatment variables	Estimate	95% CI	*p*‐value
***UDI‐6***			
Age	0.005	(−0.26, 0.27)	0.97
UDI‐6	0.18	(−0.04 to 0.39)	0.10
Bladder neck			
Closed	Reference	(−3.00, 12.99)	0.22
Open	5.00		
ALPP			
No leak	Reference	(−10.95, 3.63)	0.33
Stress leak without catheter	−3.66		
Urethral hypermobility			
Non‐type 3	Reference	(−11.66, 8.18)	0.73
Type 3	−1.74		
Bladder compliance			
Normal	Reference	(1.24, 15.15)	0.023
Poor	8.19		
***IIQ‐7***			
Age	0.075	(−0.22, 0.36)	0.61
IIQ‐7	0.45	(0.29 to 0.61)	< 0.001
Bladder neck			
Closed	Reference	(−8.34, 9.22)	0.92
Open	0.44		
ALPP			
No leak	Reference	(−10.78, 5.23)	0.50
Stress leak without catheter	−2.78		
Urethral hypermobility			
Non‐type 3	Reference	(−12.65, 8.91)	0.74
Type 3	−1.87		
Bladder compliance			
Normal	Reference	(−0.40, 14.43)	0.067
Poor	7.01		
***ICIQ‐SF***			
Age	0.077	(0.009, 0.15)	0.026
Bladder neck			
Closed	Reference	(−2.80, 1.46)	0.53
Open	−0.67		
ALPP			
No leak	Reference	(−2.37, 1.52)	0.67
Stress leak without catheter	−0.43		
Urethral hypermobility			
Non‐type 3	Reference	(−3.71, 1.46)	0.40
Type 3	−1.13		
Bladder compliance			
Normal	Reference	(0.35, 4.03)	0.022
Poor	2.19		

Associations between treatment success and the following variables were not statistically significant (all *p* > 0.05): age, pre‐treatment UDI‐6 and IIQ‐7, bladder neck status as open or not, ALPP value, MUCP at start of fill and bladder compliance. The association between treatment success and urethral hypermobility was statistically significant and large‐sized. Women with type 3 stress incontinence were more likely to report treatment success than women with non‐type 3 stress incontinence. In other words, a fixed urethra was associated with treatment success.

Apart from pre‐treatment IIQ7 scores (*p* < 0.001), none of the associations between post‐treatment IIQ7 scores and pre‐treatment variables were statistically significant. Associations between both UDI‐6 and ICIQ‐SF scores post‐treatment and the following pre‐treatment variables were not statistically significant: bladder neck, ALPP and urethral hypermobility. However, the association between UDI‐6 and ICIQ‐SF scores, and bladder compliance were statistically significant (*p* = 0.023 and *p* = 0.022, respectively); in both cases, poor bladder compliance pre‐treatment was associated with higher self‐reported scores post‐treatment, indicating poorer post‐treatment outcomes. There was also a significant association between ICIQ‐SF scores post‐treatment and age; in this case, older age was associated with poorer self‐reported outcomes. The association between pre‐ and post‐treatment UDI‐6 scores was not statistically significant.

## 4 DISCUSSION

At a median time between first injection and current assessment of 51 months (interquartile range 23.5 to 70), 51% of women treated with polyacrylamide hydrogel injections had a successful treatment outcome based on PGI‐I scores. Women with type 3 urethral hypermobility or well‐supported urethra, were more likely to report treatment success than women with urethral hypermobility before treatment. Similarly, poor bladder compliance before treatment was associated with higher urinary symptom distress (UDI‐6 scores), higher severity and frequency of urinary incontinence (ICIQ‐SF scores) post‐treatment. Older age was associated with higher levels of self‐reported urinary frequency and severity (ICIQ‐SF scores) post‐treatment. Finally, pre‐treatment incontinence impact was significantly associated with incontinence impact (IIQ7) post‐treatment.

These risk factors for success and failure are consistent with the two types of profiles of patients offered this minimally invasive treatment. The first group have a well‐supported urethra and ongoing exertion‐related leakage despite good urethral support. The second group prone to failure are frail at the outset and are more likely to be offered a minimally invasive treatment rather than a sling procedure due to concerns about voiding efficiency, capacity to undergo anaesthesia and other risks associated with frailty.

The pathophysiology of SUI is complex and postulated to be due to two main pathologic processes. One is urethral hypermobility from defective urethral support.^2^ The other a loss of proximal urethral closing function as a result of defective urethral mucosal coaptation, known also as intrinsic sphincter deficiency (ISD).^3^ Although the two are non‐mutually exclusive, a bulking agent is used to improve proximal urethral closure which is likely more effective when the urethra is well supported. Some studies suggest that polyacrylamide hydrogel treatment is suitable for treating all forms of SUI.^13^, ^14^ Giammo et al. found no significant association between urethral mobility and clinical outcome.^13^ Similarly, in a small study by Vecchioli‐Scaldazza et al. comparing seven patients with urethral hypermobility to 13 patients without urethral hypermobility, they found no difference in polyacrylamide hydrogel treatment outcomes between the two groups.^14^ Both studies evaluated the presence of urethral mobility via translabial ultrasound and defined urethral hypermobility as having a rotational angle of greater than 28 degrees between the urethra and bladder neck during Valsalva manoeuvre compared with at rest. Urethral hypermobility was not further sub‐typed in either study.

Although type 3 stress incontinence was associated with a successful outcome, the majority of women in our study had urethral hypermobility, with approximately half of these women reporting a successful outcome. Given the low risk of the procedure, these data aid in counselling women with a hypermobile urethra regarding their chance of success. In those who do not respond sufficiently to primary polyacrylamide hydrogel treatment, results may then be complemented with further sling surgery. Further work correlating the degree of urethral hypermobility and long‐term outcome of polyacrylamide hydrogel is warranted.

The severity of pre‐treatment incontinence was found to be a factor associated with post‐treatment outcome in our study, with worse incontinence impact (IIQ‐7 scores) pre‐treatment associated with worse incontinence impact after treatment. This is consistent with findings by Giammo et al. and Elmelund et al. who reported that increasing severity and frequency of baseline incontinence respectively was associated with poorer outcomes following polyacrylamide hydrogel treatment.^13^, ^15^

In a study of 97 patients treated with polyacrylamide hydrogel injection at a median follow‐up time of 15 months, Giammo et al. reported that patients with mild to moderate incontinence (defined as less than 200 g per day on 24‐h pad test) were more likely to have treatment success.^13^ Elmedlund et al.'s study consisting of 191 patients treated with polyacrylamide hydrogel and 100 patients treated with collagen injections, found that patients over the age of 60 and with less than 2.5 daily SUI episodes were associated with a cure at 12 months.^15^

There was a significant association between age and ICIQ‐SF scores after treatment. This is possibly explained by the fact that elderly patients are more likely to have mixed urinary incontinence as compared to younger patients. Elmelund et al. however reported that age greater than 60 years is associated with treatment success.^15^ This study was of a younger cohort with a mean age of 58.8 years compared to our cohort with a mean age of 63.1 years. It has been proposed that age and co‐morbidities are thought to influence the patients' perception of SUI improvement.^13^ It is possible that elderly patients may be less active and more accepting of an improvement in quality of life as a treatment goal.^13^ Younger patients may be more willing to undergo more invasive surgery^16^ for a treatment that will help optimise their fitness long term.

Poor bladder compliance was associated with poorer patient‐reported outcomes at follow‐up assessment. Bladder compliance refers to the viscoelastic behaviour of the urinary bladder, with decreasing bladder compliance associated with detrusor overactivity.^17^, ^18^ Consistent with our results, poor bladder compliance and detrusor overactivity have previously been reported to be associated with a significantly lower success rate with collagen injections.^16^, ^19^ Giammo et al., however, found no difference in polyacrylamide hydrogel treatment outcomes in patients with concomitant urgency.^13^ A randomised controlled trial (RCT) by Maher et al. reported a higher risk of de novo overactive bladder following pubovaginal slings compared with Macroplastique® injections (4.8% vs. 0%, respectively).^20^ This is in contrast to the RCT by Itkonen‐Freitas et al., whereby a higher incidence of de novo urgency of 9.3% was reported in the polyacrylamide hydrogel group versus 5.9% in the transvaginal tape group.^21^ The differences in both these studies were not statistically significant, however. The severity of co‐existing detrusor abnormalities may adversely affect judgements about the effect of an SUI treatment. It has been suggested that the coexistence of poor bladder compliance should not preclude the offering of a bulking agent for concomitant SUI given medical and other therapies can be offered.^16^ Nevertheless, in counselling for the procedure, co‐existing bladder dysfunction should temper expectations of the outcome. Given a 9.3% incidence of de novo urgency has been documented,^21^ long‐term data on bladder function following urethral bulking injections is required. This cross‐sectional study highlights the importance of ongoing care for detrusor function once the SUI component has been treated. Improved follow up of detrusor disorders would likely have the effect of improving the apparent success rate of the bulking agent.

The strength of this study lies in it being a well characterised series on long‐term polyacrylamide hydrogel only treatment outcomes performed by a single urologist.^12^ There are several limitations, however. Firstly, this is a cross‐sectional study, with pre‐treatment outcome data reliant on the accuracy and completeness of clinical records. Patients who did not respond sufficiently to primary polyacrylamide hydrogel treatment and proceeded to alternative SUI treatment were excluded from the final assessment. Clinicians should take this into consideration when interpreting the results of this study as it would also have the tendency to under‐estimate the benefits of injection therapy, most going on to have an excellent result in combination with sling surgery.

Ideally, follow‐up would be undertaken prospectively at pre‐specified time points to more accurately date the timing of treatment failure. This study demonstrated treatment failure was not associated with duration of time from the procedure to the time of follow‐up. Furthermore, logistic regression models adjusted for time since initial surgery were compatible with results from the unadjusted models. This is of no minor therapeutic significance. There has been a stated perception that bulking agent injections progressively deteriorate over time and are not durable. Anecdotal evidence from the treating urologist's experience of excision of the agent at the time of implantation of an artificial urinary sphincter demonstrated the gel unchanged at a 3‐year interval following injection. Although the findings from this study suggest polyacrylamide gel treatment may be durable if it works, the importance of systematic long‐term studies should be emphasised.

## 5 CONCLUSION

Careful selection and counselling of patients for urethral bulking with polyacrylamide hydrogel is important in order to optimise treatment outcomes and patient satisfaction. Women with type 3 stress incontinence pre‐treatment were more likely to report treatment success (PGI‐I), whereas severity of pre‐treatment incontinence, poor bladder compliance and older age were associated with poorer, self‐reported treatment outcomes. Though polyacrylamide hydrogel is complemented by sling surgery, as a standalone therapy, this study demonstrates that long‐term efficacy appears to hold in those who respond initially.

## CONFLICTS OF INTEREST

The authors have no competing interests.

## AUTHOR CONTRIBUTIONS

Study conception and design: VH, HY, HOC. Acquisition of data: VH. Analysis and interpretation: VH, HY, KG, HOC. Drafting of manuscript: VH. Critical revision: VH, HY, KG, HOC.
